# Dyadic Predictors of Child Body Shame in a Polish and Italian Sample

**DOI:** 10.3390/ijerph19148659

**Published:** 2022-07-16

**Authors:** Kamila Czepczor-Bernat, Justyna Modrzejewska, Adriana Modrzejewska, Emanuela Calandri, Silvia Gattino, Chiara Rollero

**Affiliations:** 1 Institute of Psychology, University of Wrocław, 50-527 Wroclaw, Poland; 2 Institute of Pedagogy, University of Bielsko-Biala, 43-309 Bielsko-Biala, Poland; jmodrzejewska@ath.bielsko.pl; 3 Department of Psychology, School of Health Sciences in Katowice, Medical University of Silesia in Katowice, 40-055 Katowice, Poland; adriana.modrzejewska@sum.edu.pl; 4 Department of Psychology, University of Turin, 10124 Torino, Italy; emanuela.calandri@unito.it (E.C.); silvia.gattino@unito.it (S.G.); chiara.rollero@unito.it (C.R.)

**Keywords:** child body shame, perfectionism, body dissatisfaction, parent-child dyad, Poland, Italy

## Abstract

The present study aimed at assessing the predictors (related to the functioning of a parent-child dyad) of child body shame. Therefore, in the main analysis we examined relationships among child body shame, child perfectionism, child body dissatisfaction, parent body shame, parent perfectionism, and parent body dissatisfaction. In our main hypothesis we assumed that higher levels of the abovementioned parent functioning-related variables would be associated with higher child body shame after accounting for the effects of the foregoing child functioning-related variables. The analysis finally included complete data from 420 participants, i.e., a 115 Polish and 95 Italian parent-child dyad. Participants completed: (a) child: the Objectified Body Consciousness Scale for Youth, the Child-Adolescent Perfectionism Scale, the Children’s Body Image Scale/the Figure Rating Scale; (b) parent: the Objectified Body Consciousness Scale, the Frost Multidimensional Perfectionism Scale, and the Contour Drawing Rating Scale. The results of a correlational analysis show that in both the Polish and Italian samples, the higher the level of child body shame, the higher the level of the following variables: child perfectionism, child body dissatisfaction, parent perfectionism, and parent body dissatisfaction. Interestingly, the only insignificant relationship in both samples is the association between body shame in both members of the child-parent dyad. Moreover, all steps of the regressions were significant in both Polish and Italian samples. It turned out that only in the Italian sample were all predictors significantly associated with a child’s body shame (in the Polish sample there was no significant association between child’s body shame and parent’s perfectionism). To sum up, the above studies show the importance of considering the functioning of the parent-child dyad in understanding child body shame. These findings suggest that parents’ attitudes toward their bodies and their beliefs about an ideal self should be taken into account when planning interventions to improve children’s and adolescents’ attitudes toward their bodies. This is so because it is possible for children to internalize their parents’ beliefs about how to look and how critical one should be of themselves, which can result in strong body shame when they are not perfect enough against the internalized ideal. Therefore, it is also necessary to make parents aware that children’s attitude toward their body is often a reflection of parents’ attitude toward the body.

## 1. Introduction

As is well known, our body image develops under the influence of many factors, including direct and indirect parental influences, and children have a very similar attitude towards their own body to that of their parents [1,2,3,4,5]. In the literature, there are many theoretical models involving the shaping of body image, and one of them is the Tripartite Influence Model [1]. This is a multifactorial model that proposes three main sources for the development of body image and eating disorders: parents, peers, and the media. This model not only explains in general terms the role of the sociocultural environment in body image, but also allows for the understanding of the importance of mechanisms such as modelling of weight beliefs, negative verbal comments, and internalisation in shaping the child’s body image [1]. Therefore, in order to analyse the psychological functioning of children and adolescents in relation to body image in greater depth, an increasing number of studies have recently been published in which parent-child dyads have been taken into account [3,6,7,8,9,10,11]. These studies are important, inter alia, because research shows that the effect of the negative influence of parents on the shaping of a maladaptive attitude towards the body in their child is of great importance not only in childhood and adolescence, but also in adulthood [12,13]. Importantly, many of the cited studies focused on the level of body dissatisfaction and on significant predictors of this dissatisfaction (e.g., parental comments about children’s bodies and eating behaviours [1,3,10,12,14,15]. However, to date (to the best knowledge of the authors of this article), none of the studies has focused directly on what the significant predictors of child body shame are, taking into account the psychological functioning of the parent-child dyad. Body-related shame arises inter alia based on a comparison with sociocultural standards of the body and the perception of failure to meet these standards [16]. It is related to the feeling of being imperfect and the emergence of a feeling of embarrassment with one’s body [16]. In this regard, focusing on body-related shame seems to be very important, as it seems to be crucial for the development of depression, eating disorders, and sexual dysfunction [17,18].

The variables associated with body shame are body dissatisfaction and perfectionism. The former is related to persistent negative feelings and thoughts about one’s body when it is not consistent with sociocultural standards [19], while perfectionism is often associated with unrealistically high expectations and overly critical self-assessments [20]. With reference to the abovementioned outcomes, earlier studies indicated that: (a) body dissatisfaction and body image avoidant behaviours are strongly related to body shame [21,22,23,24]; and (b) the greater the maladaptive perfectionism, the higher the negative body image [25,26]. Referring to point b above, it is worth noting that research so far has not focused directly on the relationship between perfectionism and body shame, and as we know, unrealistic expectations held by children and adolescents and an overly critical attitude toward oneself may significantly disturb their psychological functioning [27,28]. However, it can be concluded that a negative cognitive attitude toward one’s body may promote the tendency to avoid dealing with one’s body as well as increase negative emotions toward one’s body (including fear and shame), and that the at-risk group may be people with a strong perfectionist tendency (for whom it is extremely important to meet societal expectations and achieve set standards in many areas of life) [1,21,22,23,24,25,26]. Importantly, however, most of the abovementioned studies have been conducted in adults and have not considered the dyadic perspective in explaining child and adolescent functioning. Analysing similar relationships in younger groups seems to be extremely important, because research clearly shows that a negative attitude towards the body promotes many maladaptive behaviours (e.g., self-harm; [29]) and the development of various mental disorders (e.g., depression, eating disorders; [17,18,30]). Moreover, the dyadic perspective allows for a much wider view of the emergence of various difficulties in children and adolescents and, in relation to the observed difficulties, for the design of more effective preventive measures and treatments [31,32].

Taking into account the literature described above especially [1,20,21,22,23,24,25,26,27,28,30,31,32], the present study aimed at assessing the predictors (related to the psychological functioning of a child-parent dyad) of child body shame. Therefore, in the main analysis we examined relationships between child body shame, child perfectionism, child body dissatisfaction, parent body shame, parent perfectionism, and parent body dissatisfaction. In our main hypothesis we assumed that higher levels of the abovementioned parent functioning-related variables would be associated with higher child body shame after accounting for the effects of the foregoing child functioning-related variables. Moreover, in addition to the analyzes related to the main hypothesis, an exploratory analysis was also performed. This analysis was performed to assess whether child age could be a significant moderator of the relationship between parent-related body shame and child-related body shame. It was assumed that it was worth checking additionally whether, as children develop, their emotional awareness also increases (which is related to, for example the ability to recognize and make sense not only of one’s emotions, but also of other people’s), thus the emotions felt by parents towards the body (e.g., shame) are more carefully perceived and interpreted by children and therefore these emotions and related negative thoughts concerning one’s body can be strongly internalized [33,34,35,36]. It is also worth adding that in childhood and adolescence the emotions linked to the body take on greater intensity and are increasingly charged with meanings connected with the image of oneself. In conducting the above analyses, it was decided to take into account data from separate samples from Poland and Italy, as many of the previous studies indicate that the cultural and social context may be important for prevailing attractiveness standards of attractiveness and parental attitudes [37,38,39,40,41,42]. To the authors’ knowledge, there are still no comparative studies on the body image of children and their parents in a Polish and an Italian sample. Our observations and conclusions indicate that attitudes toward the body may be more liberal in Italy, which is associated with a higher degree of sexualization (self-objectification). According to our observations (which also relate to psychotherapeutic work in these two countries), Italian culture probably places more emphasis on aesthetic considerations and places more emphasis on conforming appearance to social ideals. However, scientific research is needed to verify our observations and conclusions. It is therefore worth examining whether the different socio-cultural context of the Polish and Italian samples will also influence the results we obtained.

## 2. Materials and Methods

### 2.1. Participants and Procedure

This dyadic cross-sectional study was approved by the University Research Ethics Committee (no. 04/02/2020) and was conducted in accordance with the Helsinki Declaration.

Participants were recruited via paper and online flyers (e.g., social media networks, universities, various workplaces and school) from February 2020 to September 2021 in Poland and in Italy. We used a voluntary method of sampling (a non-probability sampling technique); any of the parents (mother or father) and their child could apply for the study. Participants interested in the study contacted the researchers (by e-mail or in direct contact) and were included in the study. Participants were not remunerated for their participation. The analysis finally included complete data from 420 participants, i.e., 115 Polish and 95 Italian parent-child dyads. Table 1 shows the detailed characteristics of the group.

### 2.2. Measures

All children and parents were informed that their participation was anonymous and voluntary. All participants were told that the main goal of the study was to assess the psychosocial functioning of parents and children, and that the studies were solely questionnaire-based and concerned such aspects as: body image, self-objectification, perfectionism, and sociodemographic data. The order in which funds were presented to participants was the same as the order in which funds were presented below. Immediately prior to data collection (before completing the questionnaires), the parents signed (online) the informed consent form and the parents’ permission form and the children signed the assent form. Those who gave their informed consent to participate in the study received a link to the online survey (populated on Google Forms).

#### 2.2.1. Body Shame

Body shame was assessed with the Objectified Body Consciousness Scale for Youth (OBC-Y; [43]) and for parents with Objectified Body Consciousness Scale (OBCS; [44]). OBC-Y is a 14-item self-report measure and OBCS a 24-item one. Both questionnaires involve three subscales related to self-objectification: body shame (example item: “I would be ashamed for people to know what I really weigh” (OBC-Y), “I feel ashamed of myself when I haven’t made the effort to look my best” (OBCS), body surveillance (example item: “During the day, I think about how I look many times” (OBC-Y), “I rarely think about how I look” (OBCS), control beliefs (example item: “I can weigh what I’m supposed to if I try hard enough”(OBC-Y), “I think a person is pretty much stuck with the looks he/she is born with” (OBCS). Participants rate their level of agreement with items on a 7-point Likert-type scale ranging from 1 (“strongly disagree”) to 7 (“strongly agree”). The higher the score, the higher the self-objectification index in each of the subscales. We used only one subscale (body shame) in our analysis. Previous research shows that these measures have adequate construct and factorial validity and reliability [43,44]. In our study, Cronbach’s alpha coefficients for body shame were also acceptable (Poland: OBC-Y = 0.85, OBCS = 0.77; Italy: OBC-Y = 0.78, OBCS = 0.81).

#### 2.2.2. Perfectionism

Perfectionism was assessed for children with the Child-Adolescent Perfectionism Scale (CAPS; [45]) and for parents with the Frost Multidimensional Perfectionism Scale (FMPS; [46,47]). CAPS is a 22-item self-report measure and FMPS is a 35-item report. CAPS involves two subscales (self-oriented and socially prescribed perfectionism; example item: “I want to be the best at everything I do”) with a 5-point scale (from “false—not at all true of me” to “very true of me”). FMPS has a general scale and four subscales (concern over mistakes and doubts about actions, excessive concern with parents’ expectations and evaluation, excessively high personal standards, concern with precision, order and organisation; example item: “If I do not set the highest standards for myself, I am likely to end up a second-rate person”) with a 5-point scale (from “strongly disagree” to “strongly agree”). The higher the score, the higher the perfectionism tendency. We used only self-oriented perfectionism (CAPS) and total score from FMPS in our analysis. Previous research shows that these measures have adequate construct and factorial validity and reliability [45,46,47]. In our study, Cronbach’s alpha coefficients were also acceptable (Poland: CAPS = 0.89, FMPS = 0.91; Italy: CAPS = 0.82, FMPS = 0.79).

#### 2.2.3. Body Dissatisfaction

Body dissatisfaction was assessed for children with the Children’s Body Image Scale and Figure Rating Scale (CBIS for child and FRS for adolescent; [48,49,50]) and for parents with the Contour Drawing Rating Scale (CDRS; [51]). CBIS and FRS contain seven children’s and adolescents’ silhouettes (ranging from the thinnest to the fattest). CDRS has nine adults’ silhouettes (ranging in the same way). Children and their parents were asked: (a) “Which silhouette reflects what you look like now?” (“real body image”); (b) “Which silhouette reflects how you would like to look?” (“ideal body image”). The level of body dissatisfaction was assessed by subtracting the number of the silhouette indicated as “real” from the number of the silhouette indicated as “ideal”. The result obtained was interpreted in such a way that the higher the discrepancy between “ideal body image” and “real body image”, the greater the body dissatisfaction. Previous research shows that these measures have adequate test-retest reliability [48,49,50,51].

### 2.3. Data Analyses

Our analyses were conducted using IBM SPSS Statistic version 26 (IBM, Armonk, NY, USA) (correlations and regression models) with PROCESS macro (moderation).

#### 2.3.1. Main Analyses

The relationships between all variables were verified by correlation analysis (Pearson’s correlation coefficient) between the Polish and Italian samples. This was also a preparatory step for regression analysis to determine if all predictors were related to the dependent variable. It was found that there was no significant relationship between child-related body shame and parent-related body shame in both samples. Therefore, parent-related body shame was not included in the regression analysis as a predictor. Hierarchical regressions were used to verify our main hypothesis separately in the Polish and Italian samples. Child body shame was as a criterion variable. As a first step, we entered child-related variables (self-oriented perfectionism–CAPS–, body dissatisfaction–CBIS, FRS) and as a second step parent-related variable (perfectionism–MPS–, body dissatisfaction–CDRS). In this way, it was possible to examine the extent to which parental perfectionism and their body dissatisfaction incrementally predicted child body shame after accounting for variance associated with the other variables (child body shame and child perfectionism) had been accounted for. All assumptions of the multiple regression analysis were met. Variance inflation factors did not exceed 10 and tolerance was higher than 0.2 [52,53].

#### 2.3.2. Exploratory Analysis

For the analysis of the effect of moderation, PROCESS v4.0 by Andrew F. Hayes with bootstrap *N* = 10,000 was used [54].

## 3. Results

### 3.1. Preliminary Analyses

Descriptive statistics (*M* and *SD*) and correlations between all analysed variables are presented in Table 2 and Table 3 for the Polish and Italian sample. Summarizing the results presented in the above tables, it was found in the Polish and Italian samples that as the child’s body shame increased, the level of all the analysed variables also increased, except for the parents’ body shame.

### 3.2. Regression Analyses

The regression analyses results are shown in Table 4 and Table 5 in a Polish and Italian sample. Summarizing the results in both samples, it turned out that only in the Italian sample were all predictors significantly associated with child body shame (in the Polish sample there was no significant relation between child body shame and parent perfectionism).

### 3.3. Exploratory Analysis: Aadditional Results of Moderation Effect

**Figure 1:**
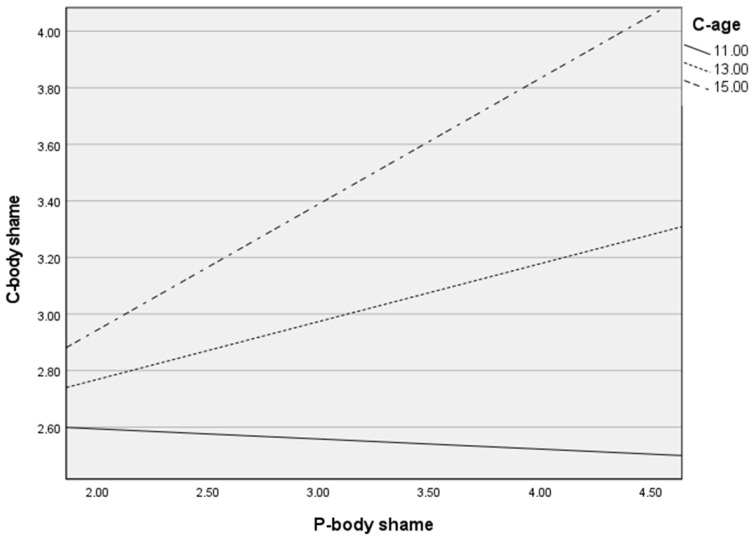
Moderation analysis: the effect of parent body shame (P-body shame) on child body shame (C-body shame) by child age (C-age) among Polish sample.

**Figure 2:**
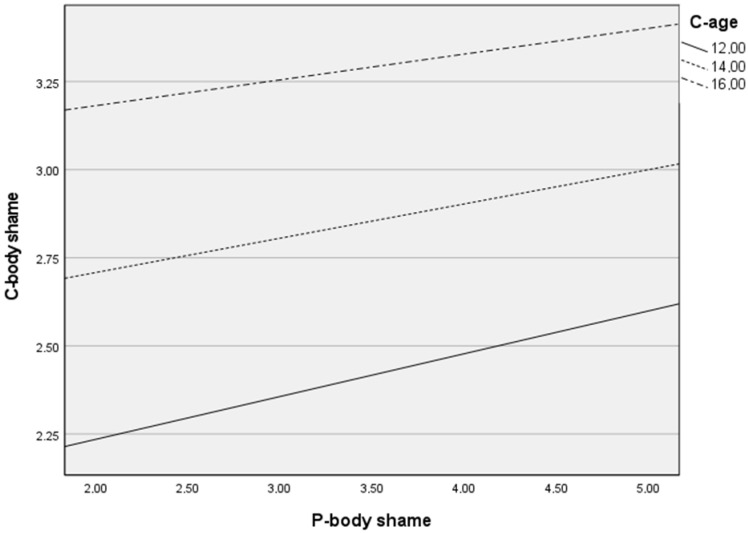
Moderation analysis: the effect of parent body shame (P-body shame) on child body shame (C-body shame) by child age (C-age) among the Italian sample.

Finally, an additional analysis was carried out to see if child age could be a significant moderator of the relationship between parent-related body shame and child-related body shame. This analysis is presented in Table 6 and Figure 1 and Figure 2. The moderation results presented in the tables and figures above show that only in the Polish sample did parents’ body shame have a positive impact on child’s body shame when children were the oldest group. No similar results were observed in the Italian sample.

## 4. Discussion

The results obtained show that in both the Polish and Italian samples, the higher the level of child body shame, the higher the level of the following variables: child perfectionism, child body dissatisfaction, parent perfectionism, and parent body dissatisfaction. Interestingly, the only insignificant relationship in both samples is the association between body shame in both members of the parent-child dyad. Previous studies suggest a general relationship between shame and perfectionism and guilt about the body [55,56]. What is worth noting here is that the literature on perfectionism is very broad and shows that we recognize many types of perfectionism [57,58]. For example, there is an association between negative perfectionism and shame and a guilt about the body, while in the case of positive perfectionism there is only a negative association with shame [57], which is consistent with our results. Also, the association of healthy perfectionism with a subjective attitude towards the body may explain our results [58]. In an attempt to explain the only insignificant relationship (child body shame and parent body shame), it can be concluded that this may be due to the role played by other variables, such as the extent to which children may be sensitive to parents’ shame (indeed, our moderation analyses showed that at least in the Polish sample, the relation between child and parent body shame is significant for the oldest children).

Regarding the main hypothesis, all steps of the regressions were significant in both Polish and Italian samples (note that parent body shame was not included in the regression analysis because there is no significant relationship between child-related body shame and parent-related body shame in both samples). The first step of the model indicated that the greater the child body shame, the higher the child perfectionism and body dissatisfaction in both samples. When parent perfectionism and parent body dissatisfaction were added to the model, the above-mentioned child variables remained significant in both Polish and Italian samples. Moreover, in the Italian sample, all predictors related to child body shame were positive. In turn, in the Polish sample, almost all predictors were significant and had a positive relationship with the dependent variable (except for parent perfectionism). This may mean that when assessing factors that are important to the child’s emotional functioning in the context of body image (and in this case in particular with regard to child body shame), in addition to taking into account the level of body dissatisfaction and the child’s perfectionism, the parent’s attitude towards one’s own body should also be taken into account. This variable is therefore a significant and moderately strong predictor of child body shame in both Polish and Italian samples. This description of the pattern of functioning is almost identical in both samples, but interestingly only in the Italian sample is parent perfectionism relevant for the prediction of child body shame. Perhaps such a situation is the result of parents modeling perfectionist behavior in accordance with the social learning model theory, which assumes that children develop perfectionist traits by observing and imitating their parents’ perfectionism [59]. It should also be remembered here that the sociocultural environment is also a factor influencing the development of perfectionism [60]. In this regard, in sociocultural research we read that the cultural values of body weight, body shape and appearance are also communicated through modeling, in particular by mothers [61]. The fact that a high level of parental perfectionism favours the internalisation of dissatisfaction with one’s body image in children could be a way to conform to beauty norms defined by the social environment in which they live and the peculiarity of this finding in the Italian sample could be due to the higher level of body sexualization in Italy. Furthermore, Italian culture places a high value on aesthetic considerations, and we can also explain the effect of parent perfectionism as a way of complying with the social environment, especially with regard to body image in Italy. However, further studies are needed to investigate the role that strict parental self-evaluation standards may play in the internalisation of children’s dissatisfaction with their own body image.

In relation to our exploratory analysis, it was only in the Polish sample that we saw that parent body shame had a positive impact on child body shame when children are the oldest group of analysed participants (+1 *SD*—15 years old). No similar results of moderation were observed in the Italian sample. Reviewing the research on parental attitudes in relation to the result obtained in the Polish sample, we read that adolescents tend to model the attitudes of their parents, especially mothers, regarding body shape [62]. Adolescents model healthy or unhealthy eating behavior, including weight management behavior [62]. It can thus be concluded that this effect can also occur in the field of body shame. In addition, similar results to those in the Italian sample were obtained in studies conducted in the United States in which the participants were teenagers aged 14–16 and their mothers. There was no association between the body shame of mothers and the body shame of adolescents [63]. It may be due to the higher levels of body sexualization in Italy. In this sense, other sources of influence, such as peers and mass media, may be more powerful for Italian youths.

## 5. Conclusions

To sum up, the studies referred to above show the importance of considering the functioning of the parent-child dyad in understanding child body shame. Referring to the tripartite influence model [1], our research results are consistent with the basic assumption of this model, which shows how important a role their parents play in developing the body image. These findings suggest that when planning interventions to improve children’s and adolescents’ attitudes toward their own bodies, parents’ attitudes toward their own bodies and their beliefs about an ideal self should also be considered. It is possible for children to internalize their parents’ beliefs about how to look and how critical one should be of themselves, which can result in strong body shame when they are not perfect enough against the internalized ideal [1,4,5]. However, further research is needed to verify this assumption using a dyadic approach. Moreover, our study has certain limitations: (a) this was a cross-sectional study, (b) all measures were self-reported, (c) a volunteer sampling technique was used, (d) the lack of analysis taking into account the gender of the child and parent, and therefore no assessment of the significance of gender compliance in both dyad members for the analysed relationships (i.e., mother-daughter, mother-son, father-son, father-daughter; [3]), (e) the exact mechanisms of formation of the child’s body image in dyadic parent-child were not analysed (e.g., modelling of weight concerns, negative verbal commentaries, internalization of socially prescribed appearance ideals), (f) some measures have not been validated in a Polish and Italian sample. These limitations should be taken into account when planning further studies. Moreover, in psychological practice, it is worth referring to: (a) the process of individuation, i.e., shaping identity consisting of developing autonomy in relations with relatives, which may lead to behavior independent of parents in the future [64], (b) developing parents’ awareness of shaping a positive body image in children and developing their ability to properly talk about the body with their children and the independence of general self-esteem from body image [65], and (c) developing awareness among parents of how important parents’ attitudes toward their own bodies are to child development, with particular attention to mechanisms such as modelling weight concerns, negative verbal comments, and the internalization of socially prescribed beauty ideals.

## Figures and Tables

**Table 1:** Demographic characteristics of parent-child dyads in Poland and Italy.

	Poland (*N* = 115)	Italy (*N* = 95)
	Child	
Gender	Female: 61 Male: 54	Female: 62 Male: 33
Age	*M* = 13.22, *SD* = 1.99	*M* = 13.86, *SD* = 1.74
Weight (kg)	*M* = 53.57, *SD* = 13.28	*M* = 52.54, *SD* = 12.25
Height (cm)	*M* = 160.87, *SD* = 11.15	*M* = 161.05, *SD* = 12.04
BMI (kg/m^2^)	*M* = 20.51, *SD* = 3.89	*M* = 20.04, *SD* = 3.04
	**Parent**	
Gender	Female: 89 Male: 26	Female: 70 Male: 25
Age	*M* = 41.57, *SD* = 6.27	*M* = 46.97, *SD* = 6.47
Weight (kg)	*M* = 73.99, *SD* = 13.22	*M* = 69.45, *SD* = 15.84
Height (cm) BMI (kg/m^2^)	*M* = 169.05, *SD* = 7.75 *M* = 25.87, *SD* = 4.16	*M* = 167.97, *SD* = 8.38 *M* = 24.48, *SD* = 4.47

**Table 2:** Descriptive statistics and correlations of parent-child dyads in Poland.

	1	2	3	4	5	6
1. Child body shame		0.43 ***	0.41 ***	0.12	0.25 *	0.27 **
2. Child perfectionism			0.02	−0.05	0.36 ***	0.04
3. Child body dissatisfaction				−0.09	0.03	0.05
4. Parent body shame					0.38 ***	0.31 ***
5. Parent perfectionism						0.09
6. Parent body dissatisfaction						
*M* ± *SD*	3.07 ± 1.58	36.27 ± 9.48	0.51 ± 1.10	3.19 ± 1.16	101.26 ± 16.74	1.84 ± 1.41

* *p* < 0.05, ** *p* < 0.01, *** *p* < 0.001.

**Table 3:** Descriptive statistics and correlations of parent-child dyads in Italy.

	1	2	3	4	5	6
1. Child body shame		0.50 ***	0.43 ***	0.08	0.26 *	0.23 *
2. Child perfectionism			0.13	−0.09	0.09	−0.08
3. Child body dissatisfaction				0.01	0.14	0.19
4. Parent body shame					0.38 ***	0.30 **
5. Parent perfectionism						−0.01
6. Parent body dissatisfaction						
*M* ± *SD*	2.82 ± 1.34	37.00 ± 8.27	0.45 ± 1.08	3.42 ± 1.21	100.46 ± 24.56	1.47 ± 1.32

* *p* < 0.05, ** *p* < 0.01, *** *p* < 0.001.

**Table 4:** Results of hierarchical regression analysis for the prediction of child body shame in Poland.

		Child Body Shame				
Step	Variables	B	SE	*β*	*t*	*p*
1		*F*(2, 114) = 29.36, *p* < 0.001, Adj. *R*^2^ = 0.33				
	Child body dissatisfaction	0.57	0.11	0.40	5.19	<0.001
	Child perfectionism	0.07	0.01	0.42	5.52	<0.001
2		*F*(4, 114) = 18.56, *p* < 0.001, Adj. *R*^2^ = 0.38 (Δ*F p* < 0.01)				
	Child body dissatisfaction	0.55	0.11	0.38	5.20	<0.001
	Child perfectionism	0.06	0.01	0.38	4.87	<0.001
	Parent body dissatisfaction	0.25	0.08	0.22	3.03	0.003
	Parent perfectionism	0.01	0.01	0.09	1.07	0.287

**Table 5:** Results of hierarchical regression analysis for the prediction of child body shame in Italy.

		Child Body Shame				
Step	Variables	B	SE	*β*	*t*	*p*
1		*F*(2, 94) = 29.38, *p* < 0.001, Adj. *R*^2^ = 0.37				
	Child body dissatisfaction	0.47	0.10	0.38	4.57	<0.001
	Child perfectionism	0.07	0.01	0.45	5.51	<0.001
2		*F*(4, 94) = 19.39, *p* < 0.001, Adj. *R*^2^ = 0.44 (Δ*F p* < 0.01)				
	Child body dissatisfaction	0.38	0.10	0.31	3.85	<0.001
	Child perfectionism	0.08	0.01	0.46	5.88	<0.001
	Parent body dissatisfaction	0.22	0.08	0.21	2.67	0.009
	Parent perfectionism	0.01	0.004	0.18	2.31	0.023

**Table 6:** Moderation analysis: results of models summaries (I) and tests of higher orders of unconditional interactions (II).

Model	
**Poland**	
X = parent body shame Y = child body shame W = child age	I: *R* = 0.37, *F* (3, 111) = 5.73, *p* < 0.01, *MSE* = 2.22, *R*^2^*-chng* = 0.03 II: *F* (1, 111) = 3.70, *p* < 0.05
**Italy**	
X = parent body shame Y = child body shame W = child age	I: *R* = 0.30, *F* (3, 91) = 2.96, *p* < 0.05, *MSE* = 1.70, *R*^2^*-chng* = 0.0001 II: *F* (1, 91) = 0.04, *p* > 0.05

X—independent variable, Y—dependent variable, W—moderator.

## Data Availability

The data that support the findings of this study are available from the corresponding author upon reasonable request.
